# Effects of Plasma Albumin on the Pharmacokinetics of Esomeprazole in ICU Patients

**DOI:** 10.1155/2018/6374374

**Published:** 2018-12-16

**Authors:** Hsin Tian, Yanyan Xu, Jiayan Wang, Weiqiang Tian, Jian Sun, Tao Zhang, Qingyun Zhou, Chuxiao Shao

**Affiliations:** ^1^Department of Intensive Care Medicine, Lishui Hospital of Zhejiang University, Lishui, Zhejiang 323000, China; ^2^Department of Pharmacy, Lishui Hospital of Zhejiang University, Lishui, Zhejiang 323000, China; ^3^Department of General Surgery, Lishui Hospital of Zhejiang University, Lishui, Zhejiang 323000, China

## Abstract

**Objectives:**

To evaluate the effects of plasma albumin on pharmacokinetics of esomeprazole in ICU patients.

**Methods:**

This study was performed in 32 consecutive intensive care unit (ICU) patients. They were divided into two groups according to the plasma albumin levels. Nineteen patients with low plasma albumin levels (<30 g/L; male/female, 12/7) were assigned to low plasma albumin group (LPAG). Thirteen patients with plasma albumin levels >30 g/L (male/female, 9/4) were assigned to high plasma albumin group (HPAG). All patients were received intravenous (IV) of 40 mg esomeprazole in 5 min. Blood samples were collected via basilic vein at different time points and concentrations of esomeprazole were determined by UPLC-MS/MS.

**Results:**

MRT_(0-∞)_, t_1/2_, V, CL, and C_max_ between two groups were significantly difference (P<0.05). Compared with HPAG, MRT_(0-∞)_, t_1/2_, and V of esomeprazole in LPAG were increased by 1.42-fold, 1.49-fold, and 1.24-fold, respectively; the maximum drug concentration of esomeprazole in LPAG was decreased to 82.5%. AUC_(0-∞)_ of LPAG was 1.23 times than that of group B. CL in LPAG was 80% of HPAG. There was no statistical difference between the two groups of AUC_(0-∞)_ and CL.

**Conclusions:**

Some pharmacokinetic parameters of esomeprazole may be changed in ICU patients with low plasma albumin.

## 1. Introduction

Esomeprazole, the S-isomer of omeprazole, is the first single isomer proton pump inhibitor (PPI) approved to treat peptic ulcer bleeding, peptic/stomach ulcer, gastroesophageal reflux disease and Zollinger-Ellison syndrome [1, 2]. The mechanism of esomeprazole treatment of acid-related disease is similar to omeprazole. Both of them were suppresses gastric acid secretion by specifically inhibiting the H^+^/K^+^-ATPase in the gastric parietal cells [3]. However, pharmacokinetic characteristics of them were difference. Many randomized controlled trials (RCTs) and cohort and case-control studies show that esomeprazole has a higher bioavailability, better clinical efficacy, and treatment capacity for acid-related diseases than omeprazole [4–6]. Depending on the superior acid control ability and good tolerance, esomeprazole has been widely used to patients with acid-related disease including of stress-ulcer prophylaxis in China.

ICU patients have a high incidence (75-100%) of stress-related mucosal disease. The critically ill patients in ICU can get stress-related gastrointestinal mucosal damage in the 24 h after the onset of the disease. Although a small proportion of these patients will bleed, the mortality will increase significantly with complication of stress-ulcer bleeding and perforation, reaching 50-80%, which is one of the common causes of death in ICU patients [7]. Therefore, esomeprazole is commonly prescribed in ICU patients for the prophylaxis of stress-related mucosal disease.

Esomeprazole has a high plasma protein binding (~97%) according to its instructions. The distribution and metabolism of esomeprazole are strongly affected by drug-protein interactions in the blood stream [8]. As we all known, the pharmacological activity of esomeprazole was temporary lost when esomeprazole combined with protein. Hypoalbuminemia is frequently observed in ICU patients with the incidence of approximately 40% [9]. The pharmacokinetics of esomeprazole probably different in those hypoalbuminemia patients due to its unbound concentrations increased. However, there is little information on the pharmacokinetics of esomeprazole in hypoalbuminemia patients. In the present study, we measured the concentration of esomeprazole in the plasma and investigated the effects of mild hypoalbuminemia on the pharmacokinetics.

## 2. Materials and Methods

The protocol was approved by the Ethics Committee of Lishui Hospital of Zhejiang University (*Ethical Review of Clinical Research-2016-43*) and was registered in Chinese Clinical Trail Registry (ChiCTR1800018516). The investigators adhered to the Declaration of Helsinki. All patients who participated in this study provided written informed consent.

### 2.1. Patients

The prospective cohort study was performed in 32 consecutive patients in ICU wards from January to December in 2017. They were aged 18 to 89 years aimed to use esomeprazole for the prophylaxis of stress-related mucosal disease. The dosage of esomeprazole was 40 mg once a day with intravenous injection for 5 min. 19 patients had low plasma albumin levels (<30 g/L; male/female, 12/7), who were assigned to low plasma albumin group (LPAG), and the remainder with higher plasma albumin levels (>30 g/L; male/female, 9/4) were assigned to high plasma albumin group (HPAG). Exclusion criteria included lactation or pregnancy, clinical diagnosis of treat peptic ulcer bleeding, peptic/stomach ulcer, gastroesophageal reflux disease, and Zollinger-Ellison syndrome, or the dosage of esomeprazole >40mg in one day for other purposes. Patients were also excluded if they had a history of treat peptic ulcer bleeding, peptic/stomach ulcer, gastroesophageal reflux disease, and Zollinger-Ellison syndrome. These patients' characteristics are summarized in Table 1.

### 2.2. Sample Collection and Measurement

Blood samples of 1.0 mL were drawn by site nurse from basilic vein: at 3, 5, 15, and 30 minutes and 1, 2, 4, 6, 8, and 10 hours after dosing. All blood samples were stored at room temperature for 30 minutes after collection, and then centrifuged at ×1500g for 10 minutes at room temperature. Plasma was transferred to a labeled 1.5 mL Eppendorf tube and stored at −20°C until analyzed.

Plasma esomeprazole levels were determined by UPLC-MS/MS according to the validated method [10]. The analytes were separated on an Acquity UPLC BEH C18 column (2.1 × 50 mm, 1.7 *μ*m). Esomeprazole was separated by gradient elution, which consisted of mobile phase A acetonitrile and A 0.1% formic acid and 5 mM ammonium formate in water. Gradient condition was detailed as follows: total run time was 3 minutes. Initially, mobile phase A was sustained as 20% from 0 to 0.7 minutes. Then, A was reached to 80% for the 0.9 minutes. Then 80% of mobile phase A was maintained for 0.5 minutes. Next, the mobile phase A was drawn back to 20% for 0.7 minutes and equilibrated as 20% for the 2 minutes. The flow rate was 0.40 ml/min, and column was 40°C. Detection was conducted with a triple quadrupole tandem mass spectrometer equipped with positive electrospray ionization (ESI) by multiple reactions monitoring (MRM) of the transitions. The ion transitions were m/z 346.2 >198.0 for esomeprazole and m/z 285.1 > 193.1 for diazepam (internal standard).

### 2.3. CYP2C19 Genetic Analysis

Whole blood samples 4 mL were obtained from each patient and collected in EDTA-anticoagulated vacuum tubes. DNA was extracted from 200 *μ*L blood using a Blood Genomic DNA Extraction Kit Genomic DNA Kit (BaiO Technology Co., Ltd., Shanghai, China) following the manufacturer's instructions. The concentration and purity of the extracted DNA samples were calculated using NanoDrop 2000 Spectrophotometer (Thermo Fisher Scientific, Waltham, USA). The variants of the CYP2C19 gene (CYP2C19*∗*1, CYP2C19*∗*2, and CYP2C19*∗*3) were detected by a commercially available kit (BaiO Technology Co., Ltd., Shanghai, China). Based on PCR results, the patients were further divided into the wild-type group (CYP2C19*∗*1/*∗*1) and mutant group (defined by the presence of at least one loss-of-function allele, including CYP2C19*∗*1/*∗*2, CYP2C19*∗*1/*∗*3, CYP2C19*∗*2/*∗*2, CYP2C19*∗*2/*∗*3, and CYP2C19*∗*3/*∗*3). Six genotypes of CYP2C19 were classified as three metabolic phenotypes. CYP2C19 genotype of *∗*1 /*∗*1 is normal metabolizer. CYP2C19 genotype of *∗*1/*∗*2 and *∗*1/*∗*3 belong to intermediate metabolizer. CYP2C19 *∗*2 /*∗*2, *∗*2/*∗*3 and *∗*3/*∗*3 belong to poor metabolizer.

### 2.4. Pharmacokinetic Analyzes

Pharmacokinetic analyses were performed with evaluable data that from patients who were eligible for the study and had a sufficient number of data points. The areas under the concentration of esomeprazole in the plasma versus the time curve from time zero to the last quantifiable concentration (AUC_0–t_) and time zero to infinity (AUC_0–*∞*_) were determined by the log-liner trapezoidal method. The residual area after the last data point was calculated as C_last_/*λ*z, where C_last_ is the concentration at the last measurable data point and *λ*z is the terminal slope of the log plasma esomeprazole concentration-time profile. Plasma terminal half-life (t_1/2_) was calculated as ln⁡2/*λ*z. The plasma clearance (CL) was estimated as Dose/AUC_0–*∞*_. The maximum esomeprazole concentration (C_max_) and the time until maximum esomeprazole concentration (t_max_) for each patient were directly determined from the plasma esomeprazole concentration-time curves. The pharmacokinetic parameters of esomeprazole (AUC_0–t_, AUC_0–*∞*_, MRT_0–t_, MRT_0–*∞*_, CL_z_, V_z_, t_1/2_, and C_max_) were analyzed by a noncompartmental model analysis by DAS 3.2.8 (Drug and Statistics 3.2.8, Shanghai China).

### 2.5. Statistical Analysis

Data were expressed as means ± SD. All continuous variables were tested for normality by Kolmogorov-Smirnov test. The data of skewed distribution were transformed into the log-normal distribution. Statistical analysis was conducted by SPSS17.0 using Student's* t*-test. P <0.05 was considered to be significantly different between the two groups.

## 3. Results

Both patients in LPAG and HPAG were homogeneous (Table 1) in terms of age, weight, and gender. Mean plasma albumin of LPAG was 26.29±3.55 g/dL (range, 16.5-29.6 g/dL). Mean plasma albumin of HPAG was 34.82±3.05 g/dL (range, 30.3-38.5 g/dL). Mean plasma albumin levels of two groups had statistical difference.

**Figure 1:**
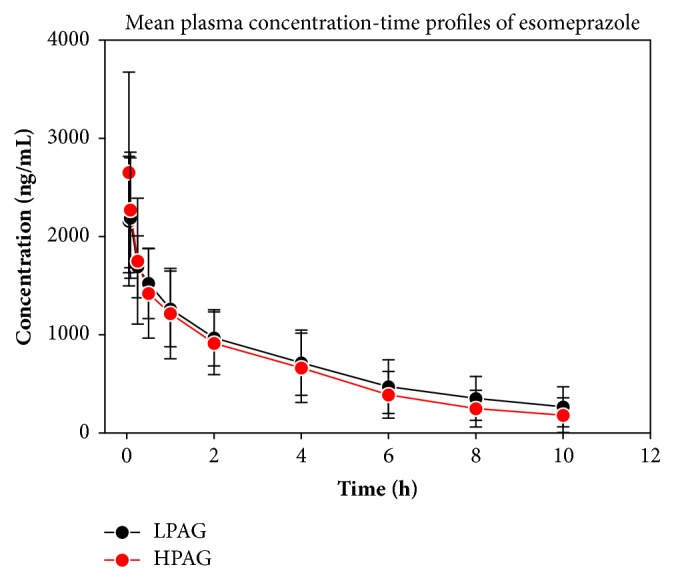
Mean plasma concentration-time profiles of esomeprazole (40mg) following intravenous injection in patients with LPAG (26.29 ±3.55, n=19) or HPAG (34.82 ±3.05, n=13).

Mean plasma concentration-time profiles of esomeprazole following intravenous injection are shown in Figure 1. Plasma esomeprazole concentration displayed high interindividual variability in both LPAG and HPAG. The coefficient of variation of mean plasma esomeprazole concentration was more than 20% in each group. From Figure 1, mean plasma esomeprazole concentration in HPAG at first three time points (3, 5, 15minutes) was higher than that in LPAG. In the next four time points, mean plasma concentration of esomeprazole was similar between two groups. However, mean plasma concentration of HPAG was lower than that of LPAG in the last three time points. The corresponding pharmacokinetic parameters of esomeprazole are shown in Table 2. Pharmacokinetic parameters of MRT_(0-*∞*)_, t_1/2_, V, CL, and C_max_ between two groups have statistical difference (P<0.05). Compared with HPAG, MRT_(0-*∞*)_, t_1/2_, and V of esomeprazole in LPAG were increased by 1.42-fold, 1.49-fold, and 1.24-fold, respectively; the maximum drug concentration of esomeprazole in LPAG was decreased to 82.5%. AUC_(0-*∞*)_ of LPAG was 1.23 times than that of HPAG. CL in LPAG was 80% of HPAG. However, AUC_(0-*∞*)_ and CL of esomeprazole were no statistical difference between the two groups (P>0.05).

## 4. Discussion

The results of this study indicated that the volume distribution (V), MRT_(0-*∞*)_, and t_1/2_ of esomeprazole were significantly increased in the patients with low plasma albumin while its C_max_ was decreased. However, the other PK parameters (e.g., AUC_(0-t)_, MRT_(0-t)_ CL, etc.) did not differ significantly between the two groups.

The combination of drug and serum albumin plays an essential role in pharmacodynamics and pharmacokinetics of drugs [8]. It is known that the free (unbound) concentration, distribution, and metabolism of various drugs are strongly affected by the combination of drug and albumin in the blood stream [11]. Hypoalbuminaemia with low plasma albumin levels is commonly found in critically ill patients due to a decrease in synthesis by the liver, an increase in albumin degradation, and/or a loss due to capillary leakage during a period of inflammation and infection [12]. Therefore, pharmacokinetics of some highly bound drugs such as warfarin, midazolam, ceftriaxone, and digitoxin may be highly altered in ICU patients with hypoalbuminemia. The volume distribution (V) is usually increased in the patients with hypoalbuminemia due to their low colloid osmotic pressure [13]. We speculated that the free esomeprazole was increased and moved to the peripheral compartment with the body fluid flow. In the present study, the V of esomeprazole in the patients of LPAG was 1.24-fold higher than that in the patients of HPAG. This result was good agreement with other studies in critically ill patients and ICU patients with hypoalbuminemia [9, 14, 15]. Changes in C_max_ due to altered plasma albumin levels can occur if the free drug in plasma and in tissue changes differently [16]. In comparison to the patients in HPAG, C_max_ was decreased to 82.5% in patients of LPAG because of the low plasma albumin level. Although the mechanism is unclear, we speculate that the decreased C_max_ of esomeprazole in patients with low plasma albumin was predominantly extracellular distribution and attributed to other factors such as volume distribution increase or oedema formation.

CL of esomeprazole did not change significantly according to the patients' plasma albumin levels. The result is similar to the previous paper by Zhang T. et al. [9]. We know that CL can calculate according to V by the equation: CL = k ×*V* [17]. It can be seen from this formula that CL is proportional to V. Our current study demonstrated large volume distribution of esomeprazole with faster clearance. However, CL was also affected by drug elimination rate constant (k). The elimination rate constant k was related to many factors, such as plasma albumin levels, the function of elimination organ, and the major esomeprazole-metabolizing enzyme CYP2C19 [18]. Meanwhile, in the noncompartmental analysis,* CL* is estimated through the AUC, which is an interim parameter that is related to the entire concentration-time curve, in which the unbalanced distribution phase is included. Additionally, in the noncompartmental analysis, the value of *V* cannot be determined directly because it is difficult to obtain the total drug amount in the body at any moment [17]. Therefore, the difference of CL with no statistical significance between LPAG and HPAG was due to the complex factors among different individuals. In view of the pharmacokinetic characteristics of esomeprazole in patients with low plasma albumin, we consider that there are three measures that can ensure better efficacy: (1) increasing the loading dose of esomeprazole; (2) the administration of intravenous injection instead of intravenous infusion which can achieve faster blood concentration; (3) shortening dosing intervals.

Half-life (t_1/2_) of esomeprazole in ICU patients with low plasma albumin levels was longer than that of healthy volunteers [19]. Compared with the pharmacokinetic parameters of esomeprazole in healthy volunteers who accept equal dosages 40 mg following intravenous (1st) [19], pharmacokinetic parameters of ICU patients with low plasma albumin were different. The half-life of healthy volunteers (0.85h) is 22.14% of ICU patients with low plasma albumin levels (3.84h) in our study. CL of healthy volunteers (17.05 L/h) is about 3-fold of ICU patients with low plasma albumin levels (5.58 L/h) in our study. C_max_ of healthy volunteers (5.53 umol/L) is 82.66% of ICU patients with low plasma albumin levels (6.69 umol/L) in our study. Combining three indicators of t_1/2_, CL, and C_max_, we can draw a conclusion that the residence time of esomeprazole in critically ill patients is significantly longer than that of healthy volunteers. We speculated that drug metabolizing enzymes like CYP2C19 inhibited esomeprazole of hepatic elimination is possible.

AUC values were associated with in patients' plasma albumin levels. For critically ill patients with hypoalbuminemia, drugs with high protein binding rate are usually eliminated quickly in vivo [13]. However, the present study demonstrated that AUC values between LPAG and HPAG had no significant difference. According to the concentration-time profiles of esomeprazole, the differences of two curves were only at the beginning 5 minutes and the last two hours of administration. This result was inconsistent with the report of dexmedetomidine with high protein binding rate by Zhang T. et al. [9]. In their paper, dexmedetomidine curve of the patients with normoalbuminuria was obviously higher than the patients with hypoalbuminemia of all time points. It is probably because esomeprazole is eliminated by hepatic metabolism mediated by CYP2C19. The genetic polymorphism of CYP2C19 leads to differences in plasma concentration among individuals.

The genetic polymorphism of CYP2C19 gene has obvious racial differences in Chinese [20]. The incidence of weak metabolisms is 15%-17% with the individual differences of CYP2C19 activity. Among the many factors may influence esomeprazole metabolism, variability of CYP2C19 genotype accounts for large percent of the PK variability [21]. As newer generations PPIs, esomeprazole suggested less influence by CYP2C19 genotype than other proton pump inhibitors, but the fraction through CYP2C19 metabolism was about 70% [21]. The PK studies showed that the AUC of esomeprazole in CYP2C19 PM phenotypes was 3-fold higher than in individuals with NM phenotypes [22]. In the current study, the proportion of PM phenotypes in HPAG was 23.08% and no patient in LPAG. This means that AUC should be higher in HPAG than LPAG. But our results were contrary to the results from previous studies [22]. Therefore, we speculated that a possible explanation was the enlarged volume distribution in the patients with low plasma albumin, which prolongs the residence time of esomeprazole in the body with the resultant changes in AUC.

There are two limitations of this study. One is small population of only Han Chinese populations patients that were included and the other is free esomeprazole that was not measured. It would be desirable to expand the scope of the different populations (white, African, etc.) and increase the sample size. Thus, the results of esomeprazole PK effect by the albumin levels may be more persuasive. The conclusion that low plasma albumin levels patients have higher concentration of free esomeprazole and lower concentration of esomeprazole with plasma protein binding could be confirmed according to the measuring free esomeprazole.

In conclusion, some pharmacokinetic parameters of esomeprazole were different between ICU patients with different plasma albumin levels. In low plasma albumin levels' patients, V, MRT_(0-*∞*)_, and t_1/2_ were increased and C_max_ decreased with significant difference.

## Figures and Tables

**Table 1:** Demographic characteristics of patients.

		LPAG (26.29 ±3.55, n=19)	HPAG (34.82 ±3.05, n=13)	P
Gender	male, n ( % )	13 (63.16)	9 (69.23)	0.72
APACHE II		20.42 (8.55)	20.23 (8.65)	0.95
Age	mean (s.d.), years	64.3 (19.1)	55.6 (20.4)	0.24
Weight	mean (s.d.), kg	61.74 (9.73)	61.92 (13.00)	0.96
Height	mean (s.d.), cm	162.89 (8.39)	166.85 (26.84)	0.19
ALT	mean (s.d.), U/L	39.58 (26.59)	52.77 (38.45)	0.34
AST	mean (s.d.), U/L	54.58 (44.49)	49.38 (41.18)	0.75
BUN	mean (s.d.), mmol/L	11.35 (9.66)	6.62 (6.19)	0.03
CCR	mean (s.d.), mL/min	76.26 (49.03)	117.19 (53.94)	0.03
Baseline hemoglobin	< 9 g / dl, n (%)	12 (57.14)	4 (30.77)	0.07
CYP2C19 normal metabolizer (NM)		9 (47.62)	4 (30.77)	0.35
CYP2C19 intermediate metabolizer (IM)		10 (47.62)	6 (46.15)	0.72
CYP2C19 poor metabolizer (PM)		0 (0.00)	3 (23.08)	/

ALT, alanine transaminase; AST, aspartate transaminase; BUN, blood urine nitrogen; CCR, creatinine clearance rate.

**Table 2:** Pharmacokinetic parameters of esomeprazole.

		LPAG (26.29 ±3.55, n=19)	HPAG (34.82 ±3.05, n=13)	P
AUC_(0-t)_	mean (s.d.), ug/mL*∗*h	6.93 (2.30)	6.42 (2.46)	0.54
AUC_(0-*∞*)_	mean (s.d.), ug/mL*∗*h	8.77 (3.70)	7.13 (3.91)	0.20
MRT_(0-t)_	mean (s.d.), h	3.14 (0.63)	2.91 (0.75)	0.34
MRT_(0-*∞*)_	mean (s.d.), h	5.39 (2.16)	3.80 (1.42)	0.03
t_1/2_	mean (s.d.), h	3.84 (1.54)	2.57 (0.99)	0.01
V	mean (s.d.), L	27.43 (7.48)	22.17 (6.02)	0.04
CL	mean (s.d.), L/h	5.58 (2.33)	6.85 (3.46)	0.21
C_max_	mean (s.d.), ug/mL	2.31 (0.50)	2.80 (0.88)	0.04

AUC, area under curve; MRT, mean retention time of the drug in the organism; t_1/2_, half-life; CL, clearance; V, volume of drug distribution; C_max_, maximum drug concentration.

## Data Availability

The data used to support the findings of this study are included within the article.
